# Multi-Omics Data Integration in Extracellular Vesicle Biology—Utopia or Future Reality?

**DOI:** 10.3390/ijms21228550

**Published:** 2020-11-13

**Authors:** Leona Chitoiu, Alexandra Dobranici, Mihaela Gherghiceanu, Sorina Dinescu, Marieta Costache

**Affiliations:** 1 Ultrastructural Pathology and Bioimaging Laboratory, ‘Victor Babeș’ National Institute of Pathology, Bucharest 050096, Romania; leona.chitoiu@ivb.ro (L.C.); mihaela.gherghiceanu@umfcd.ro (M.G.); 2 Department of Biochemistry and Molecular Biology, University of Bucharest, Bucharest 050095, Romania; dobranici.alexandra-elena@s.bio.unibuc.ro (A.D.); marieta.costache@bio.unibuc.ro (M.C.); 3 Department of Cellular, Molecular Biology and Histology, ‘Carol Davila’ University of Medicine and Pharmacy, Bucharest 050474, Romania; 4 Research Institute of the University of Bucharest, University of Bucharest, Bucharest 050663, Romania

**Keywords:** extracellular vesicles, multi-omics, data heterogeneity, data integration, systems biology

## Abstract

Extracellular vesicles (EVs) are membranous structures derived from the endosomal system or generated by plasma membrane shedding. Due to their composition of DNA, RNA, proteins, and lipids, EVs have garnered a lot of attention as an essential mechanism of cell-to-cell communication, with various implications in physiological and pathological processes. EVs are not only a highly heterogeneous population by means of size and biogenesis, but they are also a source of diverse, functionally rich biomolecules. Recent advances in high-throughput processing of biological samples have facilitated the development of databases comprised of characteristic genomic, transcriptomic, proteomic, metabolomic, and lipidomic profiles for EV cargo. Despite the in-depth approach used to map functional molecules in EV-mediated cellular cross-talk, few integrative methods have been applied to analyze the molecular interplay in these targeted delivery systems. New perspectives arise from the field of systems biology, where accounting for heterogeneity may lead to finding patterns in an apparently random pool of data. In this review, we map the biological and methodological causes of heterogeneity in EV multi-omics data and present current applications or possible statistical methods for integrating such data while keeping track of the current bottlenecks in the field.

## 1. Introduction

In the past 10 years, the scientific community’s perspective toward extracellular vesicles (EVs) has markedly focused on their role as mediators of intercellular communication, adding more insights to their known diversity and functions [1,2]. Underestimated in terms of heterogeneity, these membranous structures carry a diverse pool of functional biomolecules, most of them having unknown roles. So far, many attempts to screen these molecules or to construct reliable and reproducible molecular profiles of EV load have begun. Simultaneously, the rise of high-throughput applications in EV biology has enabled the collection of biological data at faster rates. These data are reported in various formats across a plethora of repositories, making searching and comparing results increasingly difficult. Computational methods can be used to provide integration strategies so that biological data can be observed in a system-wide manner, thus reflecting the elaborate interplay among biological variation at different levels of regulation [3].

The interest in multi-omics data integration emerges from the nature of a cell’s response to different conditions, which is best explained mechanistically when corroborating all omics levels [4]. Previous successful attempts of integrating multi-omics data have led to the characterization of informationally dense networks that accurately model human pathologies. By integrating genes and their known phenotypic profiles, groups of molecules that are involved in the same biological process (*functional modules*) have been identified [5]. Using a network-based data analysis approach, one could quantify how these modules are shared by similar diseases. According to how interconnected these modules are, disease-related genes having high association rates with a wide array of biomolecules may have a central role in the human interactome, often coding proteins acting as hubs in modulating systemic responses [6].

This review advances the idea that targeted cell-to-cell communication which occurs via EV mediation is more specific than previously believed and can be explored using multi-omics. Even though molecular cargo associated with EVs is highly heterogeneous and appears to be randomly distributed across EV subpopulations, there are putative proofs of an oriented cellular cross-talk through functional molecules. For example, tumor-derived EVs show specific integrin profiles recognized by the recipient cells in preferential metastatic locations of lung, liver, and brain cancers respectively, suggesting that EVs could prepare the pre-metastatic niche and facilitate organ-specific metastasis [7]. Here, we review existing EV multi-omics assays, highlight the biological and methodological causes of heterogeneity in EV-derived data, and present current applications or putative statistical methods for integrating such data, while keeping track of bottlenecks in this field.

## 2. Biological Diversity of EVs

EVs are membranous structures produced in the endosomal system or generated by plasma membrane shedding. They are mainly found in biological fluids and are considered a viable way of cell-to-cell communication, with various implications in physiological and pathological processes [8,9]. So far, EVs have been isolated from a large variety of biological fluids such as plasma [10], urine [11], saliva [12], cerebrospinal fluid [13], breast milk [14], ascitic fluid [15], gastric juice [16], bile [17], sputum [18], bronchoalveolar lavage fluid [19], epididymal fluid [20], and tears [21]. EVs are a highly heterogeneous population, not only by means of size and biogenesis, but also composition and biomarkers, having specific DNA, RNA, protein, lipid, and metabolite cargo. For a long period of time, size was the main characteristic used to classify EVs into exosomes and microvesicles (MVs), but contradictory results made it difficult and confusing, creating the need for alternative criteria [22,23,24,25]. As the EV biogenesis involves two main pathways that generate MVs and exosomes by membrane-trafficking processes, differentially expressed markers started being taken into consideration for a more accurate classification. Exosomes derive from the endosomal system, being synthesized as intraluminal vesicles within multivesicular bodies. Their sorting machineries are either dependent or independent of the endosomal sorting complex required for transport (ESCRT) proteins. MV generation is simpler, by plasma membrane shedding. In spite of the different packing mechanisms, the molecular profiles derived from both biogenesis processes overlap, making the MVs–exosomes dichotomy no longer useful [8,23,26] (Table 1).

EVs exert various biological roles, being involved in the immune response [37,38], regeneration [32], and as components of the extracellular matrix [39]. Early studies of EV function suggested antigen-presenting properties and the ability to stimulate T cell responses [34,40,41,42]. More recent studies indicate that EVs participate as well in autoimmune modulation processes in type 1 diabetes, as islets of Langerhans release EVs that are capable of activating peripheral blood mononuclear cells (PBMCs) [43]. In cancer, EVs are exchanged by cells within the tumor microenvironment in order to promote tumor growth and metastasis [44], vascularization [45], dormancy [46], chemoresistance [47], and metabolic reprogramming [48], mainly by transferring non-coding RNA molecules, such as miRNAs. Recently, EVs have been described as agents in modulating the spread of cancer cells toward their preferred metastases locations. Before invading other tissues and organs, tumors assure their growth by promoting the formation of a favorable micro-environment named the pre-metastatic niche (PMN) [49]. In colorectal and lung cancer, EVs enriched in small RNA species participate in activating pro-inflammatory processes through Toll-like receptor (TRL) pathways [50,51]. Other mechanisms that support metastasis are the mesothelial to mesenchymal transition and the promotion of angiogenesis through delivering miR-21-5p and miR-25-3p [52,53]. PMN formation as a result of cell-to-cell communication mediated by EVs was also observed in pancreatic, ovarian, and lung cancer [54,55,56].

As EVs play an important role in tumor growth and metastasis, it is fair to consider that they may represent therapeutic targets as well. For this matter, three strategies that might alleviate the EV contribution to tumor development were postulated: the elimination of circulating EVs based on specific surface markers, the inhibition of EV release, and the impairment of EV absorption [57]. For example, targeting the gene for the autophagy related 5 (ATG5) protein that stimulates in vivo metastasis through EV production might have promising results in holding tumor progression [58]. Drug repurposing is another promising alternative, as FDA-approved compounds such as the antibiotic sulfisoxazole impair EV secretion in breast cancer cells [59]. Taken together, EVs are of great interest not only for their crucial implications in physiological and pathological processes but also for their clinical promise.

## 3. Heterogeneous Methods and Data in EV Biology

Extensive debates on the concentration of EV cargo required mediating a biological effect or the influence of co-purified molecules (cytokines, chemokines) on assumed targets have been unfolding in the scientific community. Initially seen as questionable carriers of biomolecules [60,61], EVs now represent reliable sources of heterogeneous biological data spanning across all omics. One of the main challenges that arises even before data collection or analysis is the isolation of EVs. Most often, the difficulties stem in their small size, hydrophobic character, and low abundance in biofluids - which is usually several orders of magnitude lower than other well-known components such as albumin in blood [62].

**Figure 1:**
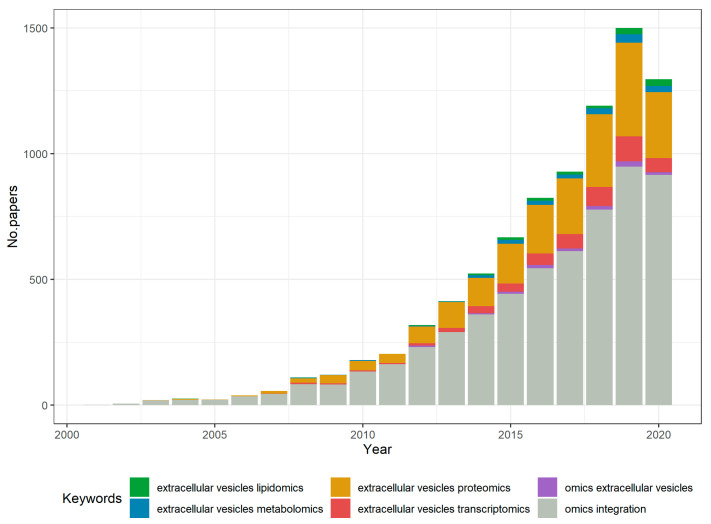
Evolution of interest in omics studies for EVs, according to the number of annual publications on PubMed. Stacked histogram created using R software [63].

In the past decade, as high-throughput technologies have been evolving, multi-omics data acquisition started to be a feasible method for EV study. Growing interest has been recorded for transcriptomics, proteomics, and metabolomics screenings, yet omics approaches for the DNA load of EVs are still difficult and costly to implement. Simultaneously, the ongoing debate around adequate bioinformatics methods to be used for such multi-level data has been focusing on integrative approaches (Figure 1).

### 3.1. Genomic Heterogeneity

**Figure 2:**
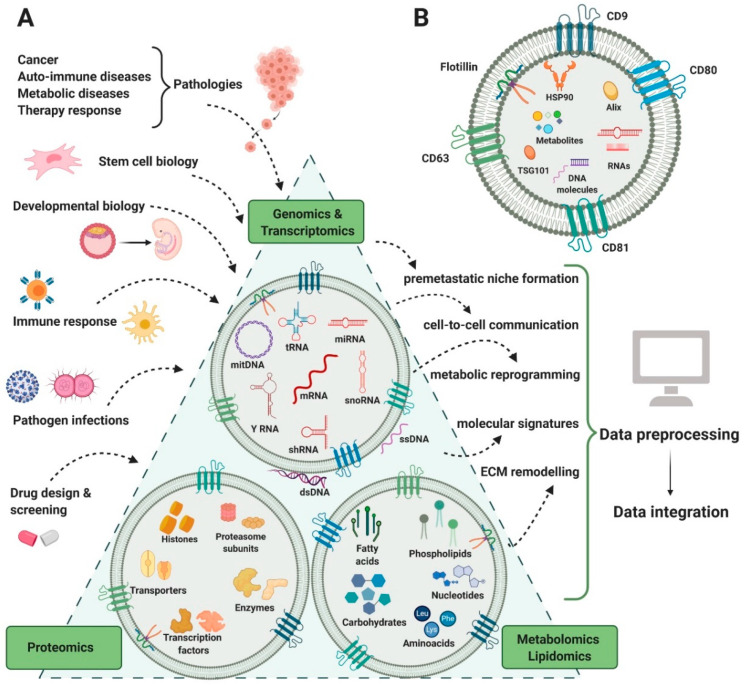
Correlation between EVs content in RNA, protein, lipid, and metabolites types and the multiple omics analyses that EV data integration can generate. Several areas of biology and medical research (input) generate complex genomics, transcriptomics, proteomics, lipidomics, and metabolomics analysis that can contribute to understanding several biological processes and functions (output). By means of bioinformatics tools, these data will be preprocessed and then integrated in order to answer various biological questions regarding intercellular communication (**A**). General structure of an EV, with specific EV markers—CD9, CD63, CD80, CD81, flotillin 1, Alix, and TSG101 (**B**). Figure created with BioRender.com.

Although EVs have been reported to carry DNA molecules ranging in length from 100 base pairs to several kilobase pairs [64], EV DNA remains poorly understood and lacks genome-wide analyses. The subject of EV DNA is still controversial due to the limited evidence for its presence outside pathological intercellular communication in cancer and immune disease [65]. So far, cardiomyocytes [66] and acinar cells of the prostate gland [67] have been reported as releasing EVs containing DNA in physiological processes. It has been proved that EVs are associated with a wide range of DNA species: single-stranded DNA (ssDNA), double-stranded DNA (dsDNA), mitochondrial DNA, and even oncogenes (c-myc) [64] (Figure 2A). Recently, whole-genome sequencing started being used in order to elucidate the identity and function of EV-transferred DNA, as it seems to cover unique regions of the genome and has differential topology [68]. However, the major limitation in preserving and isolating DNA from EVs is that the fragments seem to be preferentially oriented on the outside or on the surface of EVs, while only a small fraction is encapsulated by the lipid bilayer and protected from enzymatic degradation [68]. Even so, the possible clinical applications of characterizing EV DNA from blood and liquid biopsies seem more promising than other explored methods. For example, EV-derived DNA characterization could be preferred to circulating DNA characterization, since this last biomarker is highly unstable and represents a passive product of DNA fragmentation following cell death [69].

### 3.2. Transcriptomic Heterogeneity

A special interest has been paid to EVs transporting RNA molecules, mostly non-coding RNAs, since they modulate multiple pathways in health and disease. Most of these studies focus on malignant pathologies and try to characterize reliable RNA signatures for screening [70], diagnosis [71], or prognosis [72]. Applications in other diseases such as rheumatic heart disease, osteoarthritis, and tuberculosis [73,74,75] have been reported as well. The most popular screening method for EV-derived RNA is quantitative reverse transcriptase-PCR (qRT-PCR) due to low quantities of required samples (1 ng total RNA followed by a pre-amplification step), low costs, and straightforward data analysis that does not require bioinformatics methods. However, the major drawbacks of this technique are the limited heterogeneity of explored RNA populations due to pre-selected primers, which are often subject to bias, and the need of endogenous controls for RNA expression normalization [76]. So far, a vast panel of intravesicular RNAs has been described, comprised mostly of stable micro RNAs (miRNA), but having also representative fractions of messenger RNAs (mRNA), ribosomal RNA (rRNA), transfer RNA (tRNA), small nucleolar RNA (snoRNA), small nuclear (snRNA), long non-coding RNA (lncRNA), long intergenic non-coding RNA (lincRNA), and non-coding RNA (ncRNA) [77]. More recently, next-generation sequencing (NGS) proved to be an adaptable method for more comprehensive analyses of the RNA bestiary, showing an enrichment of EV cargo in small non-coding RNAs, such as Y-RNA, vault RNA, and signal recognition particle RNA (SRP-RNA) [78] (Figure 2A). So far, a considerable number of studies performed deep sequencing analyses on the RNA cargo of EVs isolated from human samples [79,80]. Even so, the distribution of coding and non-coding RNA can vary dramatically according to the library preparation method used for sequencing.

### 3.3. Proteomic Heterogeneity

The protein cargo of EVs is both cell and disease-type dependent, being analyzed so far using Western blotting or mass spectrometry (MS). In order to validate isolated particles such as EVs, the most popular approach is to screen samples for common markers such as CD63, CD9, CD81, TSG101, and HSP70 [81] (Figure 2B). More complex, high-throughput studies are required in order to map the heterogeneous protein cargo profiles of EVs. So far, EV proteomic profiles have been successfully characterized in both gel-based (electrophoresis setup) and gel-free (chromatographic setup) systems in mass spectrometry, shotgun proteomics, or targeted proteomics. Most often, proteins are extracted using a lysis buffer with or without detergents and are digested before the MS analysis [62]. Such attempts have been useful in evaluating the differential protein expression of EV cargo in normal cells as compared to cancer cells [82], map chemotherapy-induced variations [83], mediate tumor education and metabolic reprogramming [84], chemoresistance, and other functional adaptations of the recipient cells [85].

Although identifying the proteins present in EVs is a notable milestone for the scientific community, there are deeper layers of knowledge that require additional exploration in order to functionally understand EV proteomics. For example, post-translational modifications (PTM) such as phosphorylations, glycosilations, sumoylations, and ubiquitinations can be assayed in order to map the activation status of various proteins. These modifications can easily modulate protein conformation and function by changing the physicochemical characteristics of the protein–protein interactions (PPi) [76]. For example, the sumoylation of RNA-biding proteins responsible for molecule trafficking in EVs such as the heterogeneous nuclear ribonucleoprotein hnRNPa2B1 has been reported to facilitate selected miRNA incorporation, predominantly miR-17 and miR-93 [86].

### 3.4. Lipidomic Heterogeneity

From a structural perspective, lipids are one of the most heterogeneous classes of biomolecules due to their permutations in head groups and fatty acid chains. The limited working sample size requires in this case as well techniques with increased sensitivity in order to detect individual constituents of the EV lipidome. Even if mass spectrometers offer such high sensitivity and specificity rates, they can only provide accurate measurements of the molecular weight of the analyzed lipid, while overlaps in different classes of lipids are still posing great challenges in exact identifications [76] (Figure 2A). More recently, thin layer chromatography (TLC) coupled with mass spectrometry systems such as MALDI-TOF has also been described as an efficient method in discriminating the lipidomic content of EVs. Notably, this approach has been recently used not only to identify but also differentiate between the lipid composition of EV subpopulations [87].

Even if we expect that the lipid composition of EVs should be similar to the membrane composition of the releasing cell, direct comparison studies have shown different fractions of lipids as being enriched in EV profiles, such as glycerophospatidylcholines, glycerophospatidylethanolamines, glycerophospatidylserines, sphingomyelin, cholesterol, and ganglioside GM3, contributing to their stability and structural rigidity [88,89]. In breast cancer, there are various observations associating lipids and malignancy. EV lipidomics are also aligned to this theory, as observations on triple-negative breast cancer cell lines have shown differential lipid composition between EVs versus releasing cells, as well as tumor cells with low metastatic potential versus high metastatic potential [90].

### 3.5. Metabolomic Heterogeneity

During their biogenesis, EVs might integrate sub-nanomole concentrations of small metabolites such as carbohydrates, amino acids, nucleotides, enzymatic cofactors, and lipids. As compared to other frequent contaminants of EV lysates such as lipids and lipoproteins, metabolites are a pool of small-sized analytes; therefore, even the most sensitive targeted mass spectrometry detection systems may not be able to detect them. Hence, an enrichment in metabolites using solid phase extraction cartridges is preferred prior to spectrometry assays [76].

Despite experimental hardships that limit the study of metabolomics, EVs are already recognized as independent metabolic units that can modulate systemic changes in recipient cells [91]. For example, EVs rich in prostaglandins can be used in triggering prostaglandin-dependent pathways of inflammation in the recipient cell [92]. In contrast, the polyunsaturated fatty acids (PUFA) in EVs can serve as precursors of anti-inflammatory processes [93]. Therefore, the metabolic cargo of EVs can rewire biological pathways within cells and lead to a cascade toward pathological changes (Figure 2A).

The next big candidate for EVs screening that would complete the heterogeneous panel of EV omics is the study of their glycomes. Since glycomics would involve the identification of complex carbohydrate structures, sensitive high-throughput implementations are required. However, at the moment of writing this review, these methods have not been optimized for EV samples.

It is important to note that the omics profiles of EVs are not only diverse in terms of components, but they are also dynamic when observing disease evolution. In cancer initiation, progression, and metastasis, a comprehensive landscape of EV-derived cargo has specific functions in space (PMN formation, tumor microenvironment) and time [94]. For example, stage-specific molecular signatures of EVs can be observed in monitoring breast cancer patients as the communication in between malignant and stromal cells unfolds [95]. Metastasis due to EVs released by breast cancer cells is gradually mediated at the endothelial cell level, where angiogenesis is being promoted and the destruction of vascular endothelial barriers occurs by disruption in the formation of tight junctions [96].

## 4. Strategies of Preprocessing EV Omics Data

Following omics data acquisition, preprocessing is an essential step toward curating high-quality molecular profiles that could be used for integrative purposes. Several aspects such as accounting for contamination, using reference materials, and normalization protocols in order to correct systemic errors should represent the first stages of any bioinformatics approach. The goal of the preprocessing step should be the curation of comparable, scalable, and reproducible derived data regardless of the platform used in primary data collection, as long as the experimental conditions (health or disease status, EV source) are preserved.

Contamination in EV samples can easily occur and should be accounted before, during and after EV isolation. After being released in large amounts in a biological system, EVs represent collectively an interactive surface area that can bind various molecules also present in source biofluids, such as immunoglobulins, complement proteins, coagulation factors, lipoproteins, and cytokines in blood [97]. Depending on the viscosity of these biofluids, the putative hydrophobic interactions that involve EVs and the overlapping size and density range with many other particles, contamination with lipoproteins and protein aggregates can easily occur and interfere with the downstream analysis of EVs [98]. Purification using methods such as variations in size exclusion chromatography, ion exchange chromatography, microfiltration, or fluorescence-activated sorting can be used in order to decrease the concentration of contaminant biomolecules [25].

In order to account for contaminant levels, an alternative can be using reference materials in order to identify frequent impurities and filter them out from any further analysis. In the past years, efforts have been made in order to characterize biological reference materials for EV study, leading towards the generation of recombinant EVs (rEV) [99]. These constructs are derived from nanometer-sized immature virus-like particles produced when the gag polyprotein of HIV-1 hijacks the ESCRT pathway. Since EV budding areas are preferred by overly expressed gag polyproteins, the rEV composition resembles one of the EVs in terms of lipids and proteins. Spiking rEV in samples commonly isolated from various biofluids allows tracking of recovering efficiency, compensates for intra-method and inter-user variability induced by sample handling, and significantly improves methodological calibrations and data normalization [99].

**Figure 3:**
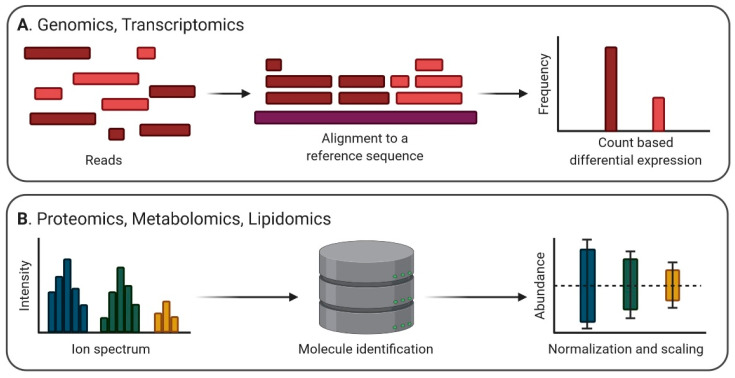
Preprocessing steps for omics data. For sequencing data, reads are curated and then aligned to a reference sequence; further on, gene and transcript quantification occurs through count-based methods (**A**). For mass spectrometry data, ion peaklists are mapped using database entries; then, identified molecule abundance is normalized for a final biomolecule profile (**B**). Figure created with BioRender.com.

For NGS, the typical normalization approach for RNA sequencing data derives from a count-based differential expression analysis. Prior to normalization, it is important to choose adaptations of data management methods based on the biases present in the dataset, which are most often enrichments in small non-coding RNAs and rRNA [100]. Data preprocessing is imperious for better sample management even before normalization: artifacts and low-quality reads corresponding to adaptors, overly represented k-mers, duplications, or contaminants should be removed using bioinformatics tools such as FastQC [101]. Further on, alignment steps using BWA [102], Bowtie [103], MAQ [104], Stampy [105], and NovoAlign [106] can be performed in order to map the reads to the reference genome. To quantify the expression level of a hit, various raw counts algorithms implemented in packages of the Bioconductor project (BaySeq, DESeq, DEXUS) can be used [107]. These algorithms estimate counts, FPKM (Fragments Per Kilobase of transcript per Million mapped reads), and TMP (Transcripts Per Million) according to the abundance of each transcript based on the alignment, whilst alignment-free implementations such as Salmon estimate TMP using pseudo-alignments in a k-mer space, according to an index built on the GRCh37 human transcriptome [108]. Moreover, additional correction steps are required for comparing transcripts within and between samples, accounting for both sequencing depth and gene length. For example, FPKM is a good measure of within-sample normalized transcript expression, yet it requires corrections for gene length when comparing changes within sample expression, as longer genes tend to accumulate more reads. On the other hand, TMP is considered to be more comparable between samples [109]. Popular algorithmic implementations of the correction methods required by differential gene expression analysis are TMM [110], PoissonSeq [111], and UpperQuartile [112]. Finally, differentially expressed genes can be the subject of gene set enrichment analysis, allowing interpretations for genome-wide expression profiles, molecular physiology, and functional genomics (Figure 3A).

In analyzing EV proteomics, one essential step is to validate the protein content as being derived exclusively from EVs and not abundant or detectable in whole cells, cellular debris, or the biological fluid used as source. For example, running a Western blot with proteins that should be absent from the sample such as histones, calnexin, or mitochondrial markers could be a good negative control prior to any further analysis involving cell culture media isolates [25]. Relative and absolute quantification for protein levels in EV samples are often made in comparison to internal standards using stable isotope labeling of amino acids in cell culture (SILAC) methods [113], or, more recently, they are based on the addition of isobaric tags in the assay samples, such as the iTRAQ labeling system [114]. For EV proteomics on serum and plasma, the presence of soluble proteins such as albumin and lipoprotein co-isolates require adaptations of the isolation methods and proteomic approach. A combination of ultracentrifugation/density cushion and size exclusion chromatography was proposed in order to better separate EVs from other lipid vesicles present in human plasma and biofluids [115]. Additionally, approaches such as micro-size exclusion chromatography for LC-MS shotgun proteomics using EV isolates from small samples (<1 mL of serum) seem more successful in untargeted characterizations, especially if two EV and lipoprotein-enriched fractions are comparatively analyzed [116]. The case of tissue-derived EV proteomic analyses is still challenging due to the difficulty of EV isolation without degrading their surface markers. So far, methods such as DNase I and collagenase treatments of tissue samples have proven successful in increasing the yield of EVs in isolates and show a minimal reduction of EV surface markers [117]. Relative protein quantification can be achieved in these cases as well by using tagged MS systems, such as the tandem mass tag technology [118]. Further on, the analysis of mass spectrometry data can be done using publicly available tools such as OpenMS [119] or LIPID MAPS (https://www.lipidmaps.org/), offering both individual or “in bulk” search algorithms for precursor ion or product ion peaklists and a wide array of statistical tools for user-uploaded data: normalization and scaling options, univariate analysis, clustering and correlation, multivariate analysis, classification and feature analysis [120]. Similarly, metabolomics mass spectrometry data can be analyzed using open source resources as HMDB (https://hmdb.ca/) for mapping annotations or pathways [121] (Figure 3).

## 5. Computational Methods for EV Data Integration

Some of the major challenges in understanding systemic effects and adaptations of biological systems such as EVs are their high dimensionality (many agents involved) and connectivity (many connections in between agents), which are often explorable by multi-omics data integration approaches. Various computational methods have been described as associating heterogeneous data in the attempt to gain comprehensive biological insights at the organism level, yet no previous studies were reported in the case of intercellular communication systems such as EVs. Integrative methods can be used to assess modulatory relationships between omics layers in order to investigate complex biological problems that have been unsolvable so far with less complex approaches. In health, one could evaluate the molecular exchange between cells within and across tissues and its role in functional adaptation to the environment. In disease, studies on the systematic response to drug treatment could be monitored using EV cargo. Furthermore, the molecular states of disease progression could be characterized so that new panels of membrane-coated, circulating biomarkers could be described and monitored in clinical applications.

As the main goal of data integration strategies has been so far the development of precision medicine models, not all current methodologies may be translatable to EV study. Some of the most popular approaches which are of potential interest in EV data integration are further analyzed in this review: correlation-based methods, network-based methods, Bayesian methods, and multi-step methods. One common characteristic of these methods is that once multiple associations point at the same molecules, false positives are less likely to occur [3]. Ideally, as heterogeneous data coming from different sources reflecting the same phenomenon are combined, the positive signals are often mutually reinforcing, while uncorrelated noise tends to cancel itself out [122].

### 5.1. Correlation-Based Methods

Believed to be some of the most approachable and popular implementations of data integration, correlation-based methods can be used in order to investigate the relationship between two sets of variables [123]. For example, correlations can be estimated between two different sets of omics recorded on the same EV samples, under the assumption of cause–effect relationships. Correlations between genomics and transcriptomics, transcriptomics and proteomics, and transcriptomics and metabolomics might be firstly investigated as they follow the logic of the central dogma of molecular biology [124]. However, the current perspective regarding the cross-talk between omics layers recognizes their bidirectional modulation; therefore, correlations between metabolomics and genomics might be of interest as well [125].

**Figure 4:**
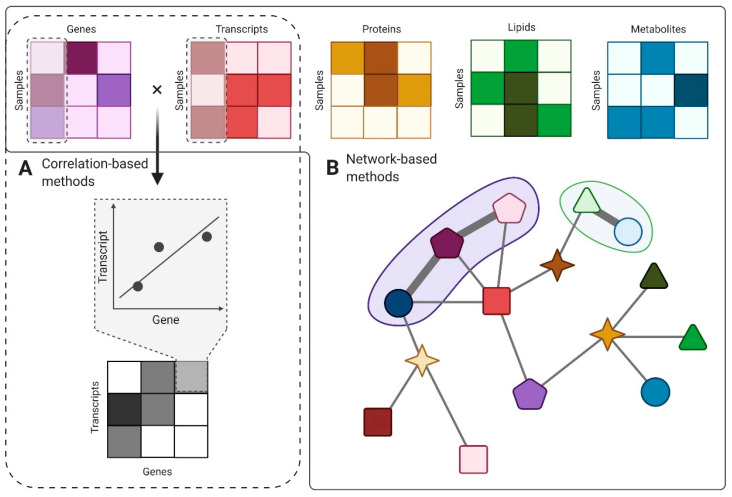
Putative strategy for EV multi-omics data integration. Multi-omics profiles can be observed from a qualitative perspective, accounting for biomolecules preferentially present or absent in certain pathophysiological states, or from a quantitative perspective, accounting for biomolecule abundancies (color gradient) and functional enrichments. Integration of multi-omics data can be achieved using correlation-based methods, a strategy endorsed for a 2 by 2 analysis of omics profiles across samples (**A**), or network-based methods, where the diversity of omics layers can be observed in graph-like structures where biomolecules are nodes and the relationships between them are weighted edges (**B**). The goal of this last approach is to identify functional modules which are activated during EV-mediated cell-to-cell communication. Figure created with BioRender.com.

Stemming in the bidimensional representation of quantified omics data as matrices, the use of mathematical operations could characterize and evaluate in terms of significance novel relationships between the recorded pairs. By multiplying two matrices that share one axis, correlations between the remaining axes can be easily achieved. A measure of the strength of the correlation can be even the color embedded in the resulting matrix in a heatmap visualization (Figure 4A). Moreover, by clustering the resulting matrix by rows and columns, one could evaluate groups that share common features in biological processes [122]. One bottleneck of this approach is the management of outliers, which can drastically influence the strength of the correlation for measures such as Pearson’s *ρ*. Alternative methods can use other rank-based correlations such as Kendall or Spearman, which are less sensitive to outliers. In some instances, outlier detection and removal approaches may be preferable, yet usually, these are not desired unless clearly advocated.

### 5.2. Network-Based Methods

Modeling complex biological interactions through the network-based method is a useful approach in EV data integration. Nodes could be represented by biomolecules (genes, transcripts, proteins, lipids from EVs cargo), and edges would define the relationships between them. Connections can be weighted according to data values or embed additional layers of information based on previous knowledge such as ontologies (standardized formulations that define the cause–effect relationships in between biomolecules, e.g., “is-a”, “is-modulated-by”, “interacts-with”). So far, approaches based on Bayesian networks that evaluate the likelihood between possible biological causes (pathologies) and their known effects (symptoms) have been successful in integrating heterogeneous, noisy data. Novel approaches put an emphasis on the topology of these networks and can be used to detect significant pathways involved in EV biology or discover network sub-clusters with functional roles [123] (Figure 4B). For example, by accounting for the internal similarity in high-throughput data and seeking sub-network modules that manifest high similarity, biologically meaningful and relevant functional modules can be obtained [126]. The major advantages of network-based methods are their resemblance to the natural interconnectivity of biological systems and the user-friendly visualization that invites systemic exploration [122].

One successful attempt of using network-based methods in EV biology is the case of discovering novel biomarkers in primary open-angle glaucoma (POAG). As a multifactorial, chronic neurodegenerative disease, POAG starts showing symptoms only in late stages. The need for predictive biomolecules that could be used in early diagnosis has led to an extensive proteomic and metabolomic screen of EV-derived cargo. Pathway analysis has correctly identified the capability of EV cargo in triggering inflammatory responses, which is an observation that was later confirmed by a genome-wide association study linking POAG etiology with endopeptidase activity in apoptotic processes [127]. Another application of network-based methods is the study of enriched EV components. By studying colorectal cancer-derived EVs, interaction networks were modeled using differentially expressed metabolites, lipids, and proteins in cell culture as compared to serum-isolated EVs. The joint networks for the two screened samples showed that fatty acid and amino acid metabolism is significantly altered in colorectal cancer [128].

Additional candidates for data integration strategies are also Bayesian methods and multi-step methods. One of the main advantages of using Bayesian methods for EV data integration is that they can be used for making assumptions on two different layers, on different types of data, as well as on the previously described correlations between those types of data [123]. Multi-step methods solve data integration in modular stages: first, they find relationships between the different data types; second, they map the connections between these relationships and a trait or phenotype of choice [3].

## 6. Challenges in EV Multi-Omics Integration

An ongoing debate in the scientific community is whether EVs have signal sorting mechanisms and represent a selective cellular communication pathway, or their cargo is randomly packed and therefore difficult to analyze in comprehensive approaches. Even though systemic perspectives can lead to a better understanding of EV functionality, as computational biology tends to bring order into chaotic aspects of life [129], various obstacles still need to be overcome.

First, the standardization of sample collection and processing is a key player in having access to reproducible datasets that could be compared for integrative purposes. As the methods used in EV biology are diverse, strategies for improving the signal-to-noise ratio need to be developed in order to prevent the accumulation of more contaminants than analytes in EV isolates. Depending on the EV isolation technique and the biofluid used as source material, contamination with proteins, lipids, chylomicrons, low-density lipoproteins, and high-density lipoproteins can easily occur [130]. Protocol optimizations are required for each study and should be adapted according to the desired EV recovery and specificity rates for the experimental hypothesis [25].

Second, improved reporting of experimental design and results is needed for consistency in the literature and reproducibility. One tool that aims to centralize knowledge in EV research and enable transparent reporting of best methodologies of EV isolation and characterization is EV-TRACK, which is a crowdsourced database created by a multinational consortium [131]. EV-TRACK records experimental guidelines in order to map the evolution of EV research, enables informed dialogs in the scientific community, and ultimately facilitates the standardization of the field, ensuring the comparability of results in a meta-analysis approach. The key feature that serves this purpose is an EV-METRIC, which is a quantifiable measure on how a study maps the ongoing EV isolation (specifications of the used separation methods, such as ultracentrifugation or density gradient), protein analysis (on both EV enriched and non-EV enriched proteins, antibody specifications, lysate preparation), and particle analysis (implementation of both qualitative methods such as electron microscopy and quantitative methods such as nanoparticle tracking analysis). Studies in the past five years show an average EV-METRIC of 41%, indicating that EV research is still deficient in transparent methodological reporting [131].

Third, the availability and consistency of previously reported data is a major limitation for validation and meta-analysis studies. Although data heterogeneity can be substantially induced by sample preparation protocols, large collections of data can yield more reproducible and biologically interpretable observations. Ultimately, such online databases developed for EV research can be used in order to validate original findings and check distributions of multi-omic profiles according to reproducible experimental setups. Often, these databases comprise of extensive transcriptomic, proteomic, metabolomic, and lipidomic datasets, standard experimental parameters in EV isolation and characterization protocols, as well as functional annotations. For example, Vesiclepedia (http://www.microvesicles.org/) is a continuously evolving database having protein, mRNA, miRNA, and lipid entries that allows users to both query and download data regarding EV cargo and enables biological pathway enrichment analyses using the FunRich plug-in [132,133]. ExoCarta (http://exocarta.org/) is a manually curated database of EV proteins, RNA, and lipids from both published and unpublished studies [134]. EVpedia (http://evpedia.info/) is a web-based database that aims to be a fundamental repository in the advancement of EV research, which provides a wide palette of tools for Gene Ontology (GO) enrichment analysis, network-based analysis for proteins and RNA, or ortholog identification [135]. More targeted databases offer even wider sets of previously mapped biomolecules in specific tissues and diseases, such as EVmiRNA (http://bioinfo.life.hust.edu.cn/EVmiRNA), which is a comprehensive gene expression database for 461 miRNAs isolated from healthy controls or cancerous tissues that are annotated with pathway information and putative targets [136].

Once challenges in acquiring and reporting single-omics data for EVs are overcome, the integration of high-throughput data is mostly limited to the degree of connectivity within and between omics layers [122]. Additionally, good quality annotations are required to observe and formulate testing hypotheses on the integrative dataset. The integration of transcriptomics data with other types of EV-wide data can facilitate a better understanding of how gene expression relates to molecular physiology. For example, the integration of RNA sequencing data and miRNA sequencing data has the potential to disclose some of the regulatory effects of miRNA on transcript levels [109]. The main limitation of this approach is poor target prediction for miRNA molecules. Several bioinformatics tools such as miRBase [137] and SePIA [138] try to overcome the need of annotations by testing significant associations between genes, miRNAs, pathways, and Gene Ontology (GO) terms. Alternatively, integrating transcriptomics data with proteomics and metabolomics is just as challenging due to the limited availability of EV-derived mRNA data and generally low correlation between these datasets, which is roughly 0.4 [139].

Analyzing the relationships between different layers of omics also has its own obstacles. For example, distinguishing confounding effects from biologically relevant mechanisms is a challenging task. Defined as variables whose presence affects the variables being studied so that results do not reflect the actual relationships among data, confounders should be looked into and properly addressed using advanced statistical models [140]. Assuming that the quantity and quality of patient-derived EVs can be affected by numerous factors including age, sex, body mass index, disease, use of medications, general lifestyle, and dietary habits, statistical methods are required to take into account these effects [141]. Ideally, in addition to a validated control normalization, further steps toward cancelling any possible confounding effects should be performed in order to minimize artifacts in EV analyses.

## 7. Conclusions

As EVs are recognized as trans-genomic agents that have emerging roles in disease evolution, applications of integrative analyses on EV-derived data are not utopic endeavors and can help us better understand the relationships between their surface markers and their cargo. Since EVs can be seen as less complex biological systems, their study could benefit from novel integrative approaches developed at the cutting-edge of computational biology, as long as standardization in EV isolation and characterization, transparent reporting, and data availability are assured. Since the interest in EV research is increasing and bioinformatics tools for normalization and processing become friendlier to users with non-computational backgrounds, we might gain more insights into the intricate cellular communication and modulation systems in the following years.

## Figures and Tables

**Table 1:** Evolution of extracellular vesicles (EV) dichotomous classification according to the accumulation of recent knowledge.

Historical Criteria	Early Knowledge	Current Knowledge
**Size**	MVs range between 100 and 1000 nm, while exosomes have dimeters smaller than 100 nm [23,27]	Classification no longer in use, MVs can be smaller than 100 nm, exosomes have an upper limit based on endosomal size (up to 150 nm or larger); “small EVs” and “medium/large EVs” nomenclature is preferred [25]
**Protein content**	Different marker profiles due to biogenesis: GTP-binding proteins (ARF6), vesicle-associated membrane protein 3 (VAMP3), proteasomes, mitochondria-related proteins for MVs, transduction or scaffolding proteins (Syntenin 1), extracellular matrix, cell adhesion, receptor binding proteins and endosome-binding proteins (TSG101) for exosomes [24,28,29]	No molecular markers that could characterize specifically each EV subtype, yet validation with three markers from three different classes is required in order to evaluate tissue specificity, lipid, or membrane-binding ability and purity [25]
**Lipid content**	Enriched contents according to the EV subtype: ceramides and sphingomyelins in MVs, cardiolipins in exosomes [29]	Lipid ratios in EVs are not yet established [25]; more studies are needed in order to compare the lipid profiles of EVs with co-isolated lipoproteins and validate characteristic EV lipid contents such as lysoglycerophospholipids [30]
**Nucleic acid content**	DNA, mRNA, ncRNA, and especially miRNA in both MVs and exosomes; origin-specific miRNA profiles for exosomes [8,31]	Confirmed specific incorporation of RNAs into subtypes of EVs [25]
**Isolation and purification methods**	Differential centrifugation or ultracentrifugation (10,000–20,000× *g* for MVs, 100,000–125,000× *g* for exosomes), size exclusion chromatography, immunoaffinity capture [32,33,34,35,36]	No “golden standard” method to isolate and/or purify EVs, the choice is to be made based on the downstream applications, recovery, and specificity rates [24,25]

## Abbreviations

DNA	deoxyribonucleic acid
dsDNA	double-stranded DNA
ESCRT	endosomal sorting complex required for transport
EVs	extracellular vesicles
FPKM	Fragments Per Kilobase of transcript per Million mapped reads
GO	Gene Ontology
lincRNA	long intergenic non-coding ribonucleic acid
lncRNA	long non-coding ribonucleic acid
miRNA	micro-ribonucleic acid
mRNA	messenger ribonucleic acid
MS	mass spectrometry
MVs	microvesicles
ncRNA	non-coding ribonucleic acid
ng	nanogram
PBMC	peripheral blood mononuclear cells
PCR	polymer chain reaction
PMN	pre-metastatic niche
POAG	primary open-angle glaucoma
PPi	protein-protein interactions
PTM	post-translational modifications
PUFA	polyunsaturated fatty acids
qRT-PCR	quantitative reverse transcriptase polymer chain reaction
rEV	recombinant extracellular vesicles
RNA	ribonucleic acid
rRNA	ribosomal ribonucleic acid
SILAC	stable isotope labeling of amino acids in cell culture
snoRNA	small nucleolar ribonucleic acid
snRNA	small nuclear ribonucleic acid
SRP-RNA	signal recognition particle ribonucleic acid
ssDNA	single-stranded DNA
TLC	thin layer chromatography
TMP	Transcripts Per Million
tRNA	transfer ribonucleic acid
